# Supplementary material for: Visualizing MiRNA Regulation of Apoptosis for Investigating the Feasibility of MiRNA-Targeted Therapy Using a Fluorescent Nanoprobe
Source: Pharmaceutics. 2022 Jun 25;14(7):1349. doi: 10.3390/pharmaceutics14071349 (PMC9323288; doi:10.3390/pharmaceutics14071349)
Supplement: Supplementary file 1 [file pharmaceutics-14-01349-s001.zip › pharmaceutics-1731836-supplementary.pdf]

pharmaceutics

MDPI

Supplementary Materials:

Visualizing MiRNA Regulation of Apoptosis for Investigating the Feasibility of MiRNA-Targeted Therapy Using a Fluorescent Nanoprobe

Mingyao Ren [1], Zhe Chen [1], Chuandong Ge [1], Wei Hu [1], Jing Xu [1], Limin Yang [2], Mingming Luan [1]* and Nianxing Wang [1]*

Experimental Details:

Materials and Instruments

Materials. Tris (hydroxymethyl) methyl aminomethane, dimethyl sulfoxide (DMSO), $CaCl_2$, chloroauric acid ($HAuCl_4$•$4H_2O$, 99.99%), sodium citrate ($C_6H_5Na_3O_7$•$2H_2O$) were purchased from China National Pharmaceutical Group Corporation (Shanghai, China); Dopamine hydrochloride and lipopolysaccharide (LPS) were purchased from Solarbio company (Beijing, China); Dithiothreitol (DTT) was purchased from Haohong Biopharmaceutical Company (Shanghai, China). Staurosporine (STS) was purchased from MedChemExpress company. Caspase-3 was purchased from Abcam company. Lipofectamine 2000 was purchased from Invitrogen company. 3-(4,5-dimethylthiazol-2)-2,5-diphenyltetrazolium bromide salt (MTT) was purchased from Sigma Chemical company. OPTI-MEM was purchased from GIBCO. DMEM, fetal bovine serum, penicillin/streptomycin and trypsin was purchased from Biological Industries. Glutathione, hemoglobin and bovine serum protein was purchased from Shanghai Sangon Biotech. A549 (the human lung cancer cell lines) and Hela (the human cervical cancer cell lines) were purchased from Procell (Wuhan, China). All aqueous solutions were purchased from Wahaha Group Corporation.

Instruments.

Absorption spectra were measured on a UV-vis spectrometer (UV-2600, SHIMADZU). Fluorescence spectra were carried out through Fluorescence Spectrometer (F97 Pro, Shanghai Prism Technology Co., Ltd.). Absorbance in the MTT assay was detected using microplate reader (Thermo Fisher Scientific). All pH measurements were measured with a digital pH-meter (pH-3c, Shanghai LeiCi, China). RT-PCR was performed on a EDC-810 (Dong Sheng Innovative Biotechnology Co. Ltd.). Transmission electron microscopy (TEM) was taken on a JEM-2100 electron microscope. Confocal fluorescence images were accomplished with a confocal laser scanning microscopy (Leica SP8, Germany).

Synthesis of Oligonucleotides and Peptide.

All DNA oligonucleotides were artificially synthesized and purified by Shanghai Sangon Biotech (Shanghai, China). All peptides were artificially synthesized and purified by Wuhan Minghao Biotechnology Co. The sequences information of oligonucleotides and peptides are showed in Table S1.

Table S1. DNA sequence and peptide information

Oligonucleotide	Sequences
ssDNA-21	5′-Cy5-TCAACATCAGTCTGATAAGCTA-3′
Target-21	5′-TAGCTTATCAGACTGATGTTGA-3′

mistarget-21	5′-TAGCATATCAGAGTGATGATGA-3′
Target-221	5′-AGCTACATTGTCTGCTGGGTTTC-3′
Target-67	5′-CGGAGTGTCAAGAGGTGTGCAGA-3′
peptide	FITC-Ahx-Gly-Gly-Asp-Glu-Val-Asp-Gly-Gly-Cys
miRNA-21 NC	5′-UUGUACUACACAAAAGUACUG-3′
miRNA-21 mimics	5′- UAGCUUAUCAGACUGAUGUUGA-3′
miRNA-21 inhibitor	5′- UCAACAUCAGUCUGAUAAGCUA-3′

Cell Culture.

All the cells were incubated in Dulbecco's modified Eagles medium which was supplemented with 1% antibiotics penicillin/streptomycin and 10% fetal bovine serum (FBS) and kept in a humidified atmosphere of 5% CO_2 at 37℃.

RT-PCR.

Total RNA was extracted from each group of cells with Trizol reagent. The synthesis of cDNA was carried out using HiScript II Q Select RT SuperMix for qPCR(+gDNA wiper). RT-PCR analysis was carried out with SYBR Green Master Mix on EDC-810. The relative level of miRNA-21 was calculated by using $2^{-\Delta\Delta Ct}$ method. U6 gene was housekeeping gene. The primers used for PCR are listed below: U6 forward: 5′-CTCGCTTCGGCAGCACA-3′, U6 reverse: 5′-AACGCTTCACGAATTTGCGT-3′. miRNA-21 forward: 5′- GTCGTATCCAGTGCAGGGTCCGAGGTATTCGCACTGGATACGACTCAACA -3′, miRNA-21 reverse: 5′- GCGCGTAGCTTATCAGACTGA -3′.

Western Blot.

The cells were washed with ice-cold PBS twice and cellular proteins were extracted in a lysis buffer (50 mM Tris-HCl, pH 7.4, 0.5% SDS, 150 mM NaCl, 1% NP40, 1% Trition, 100 mM PMSF, 5 mM $NaVO_3$, 50 mM NaF and protease inhibitor cocktail). Protein concentration was measured using BCA Protein Assay Kit (Beyotime, China). The samples were boiled in loading buffer for 5 min. Protein samples were separated on an SDS-PAGE gel and transferred on a nitrocellulose membrane (Bio-Rad) which was blocked in 5% nonfat dry milk in TBST buffer for 1 h at room temperature. Membrane was incubated overnight at 4℃ with the primary antibodies. Subsequently, membrane was incubated with peroxidase-conjugated secondary antibody for 1 h at room temperature. Finally, the mixed Enhanced Luminol Reagent and Oxidizing Reagent were added dropwise to the membrane, and the visualized enhanced chemiluminescence signals were collected using ChemiDocTMXRS (Bio-Rad).

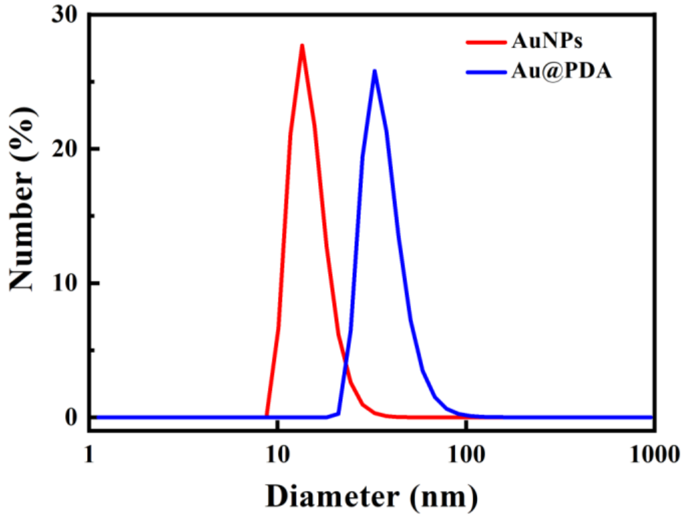

Figure S1. DLS spectra of AuNPs (red) and Au@PDA NPs (blue).

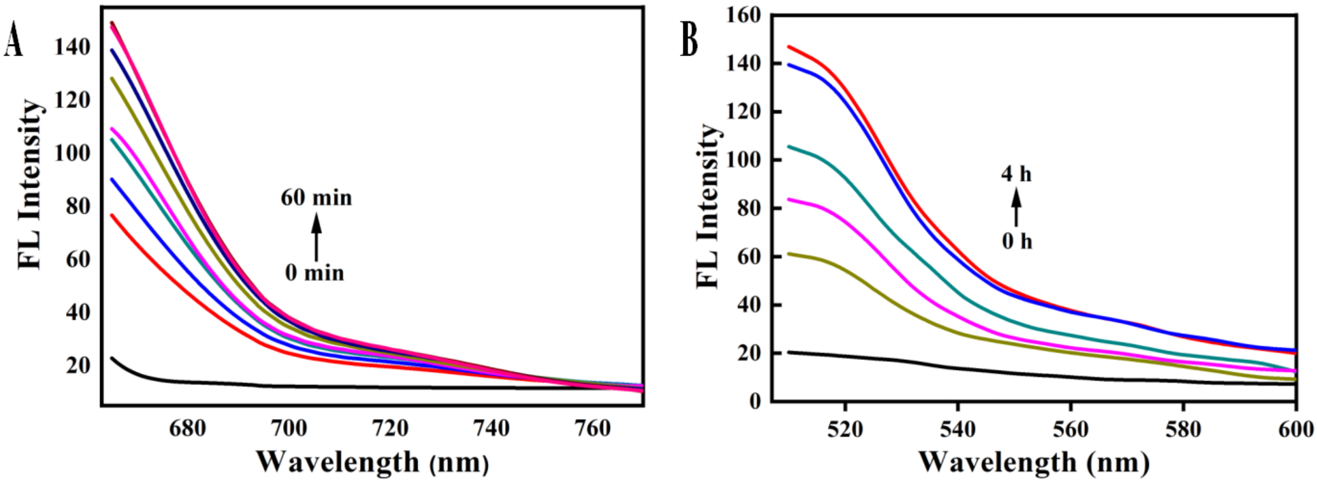

Figure S2. (A) The kinetic fluorescence spectra of the nanoprobe in the presence of miRNA-21 (A) and caspase-3 (B).

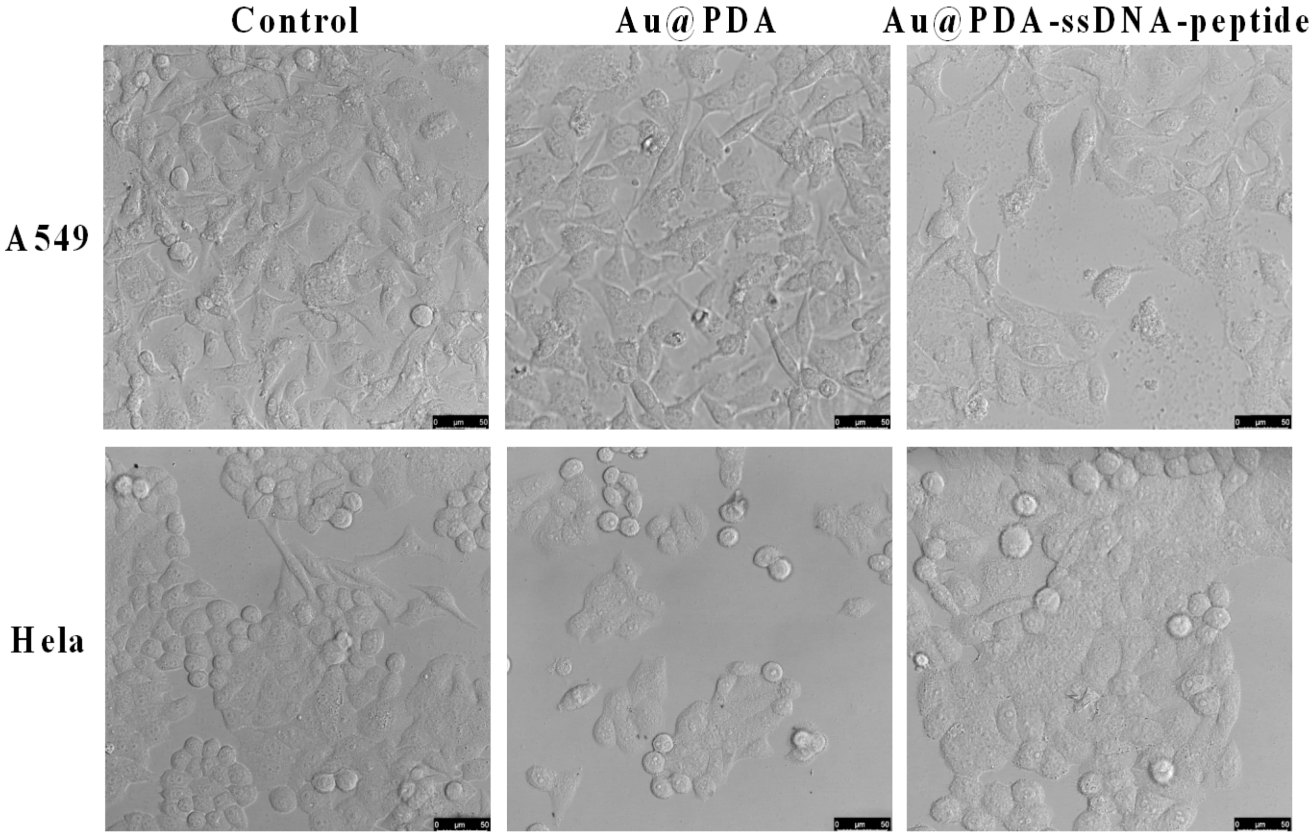

Figure S3. Bright field images of cells treated with Au@PDA NPs or Au@PDA-ssDNA-peptide NPs. Scale bar is 50 μm.

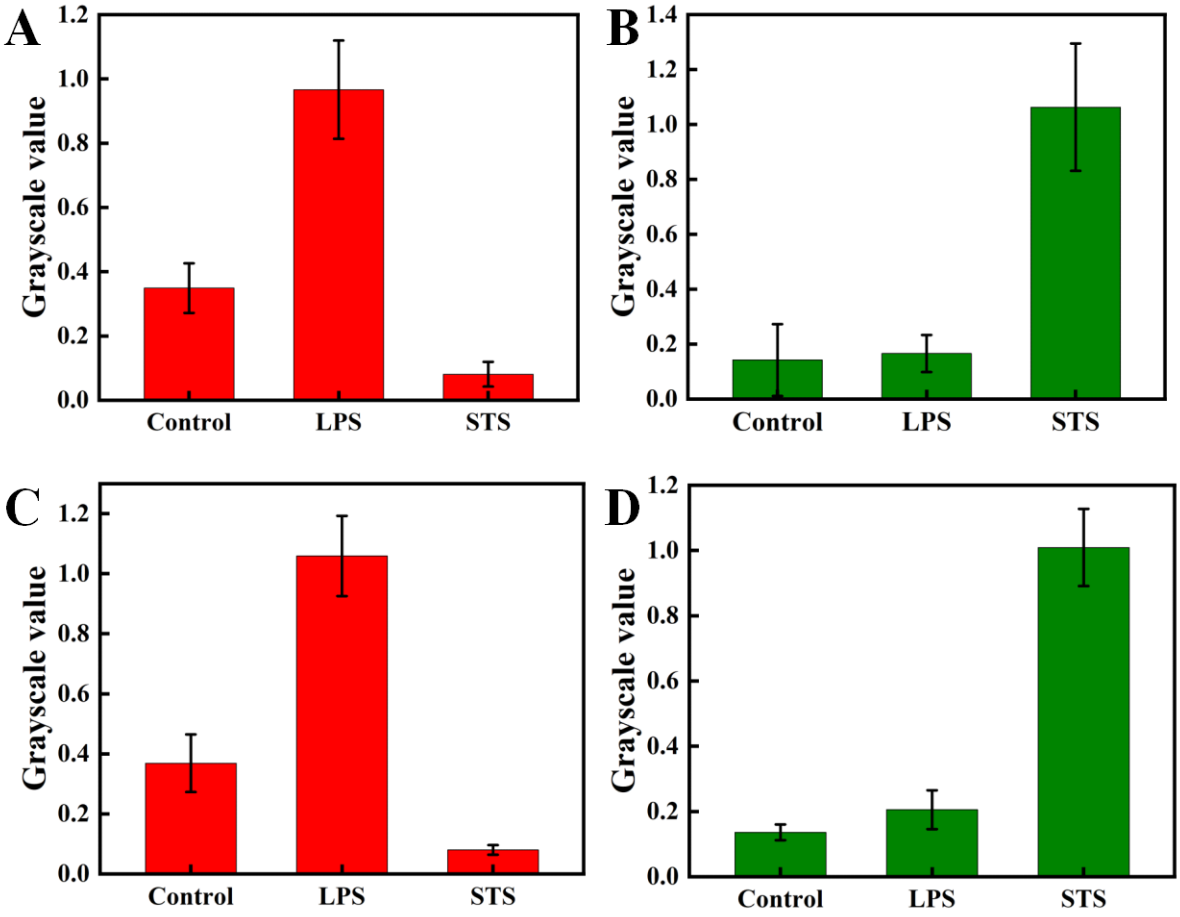

Figure S4. Grayscale values of fluorescence intensities obtained from CLSM to quantitatively reveal the different expression levels of miRNA-21 (A) and caspase-3 (B) in Hela cells after treated with different drugs. Grayscale values of fluorescence intensities obtained from CLSM to quantitatively reveal the different expression levels of miRNA-21 (C) and caspase-3 (D) in A549 cells after treated with different drugs.

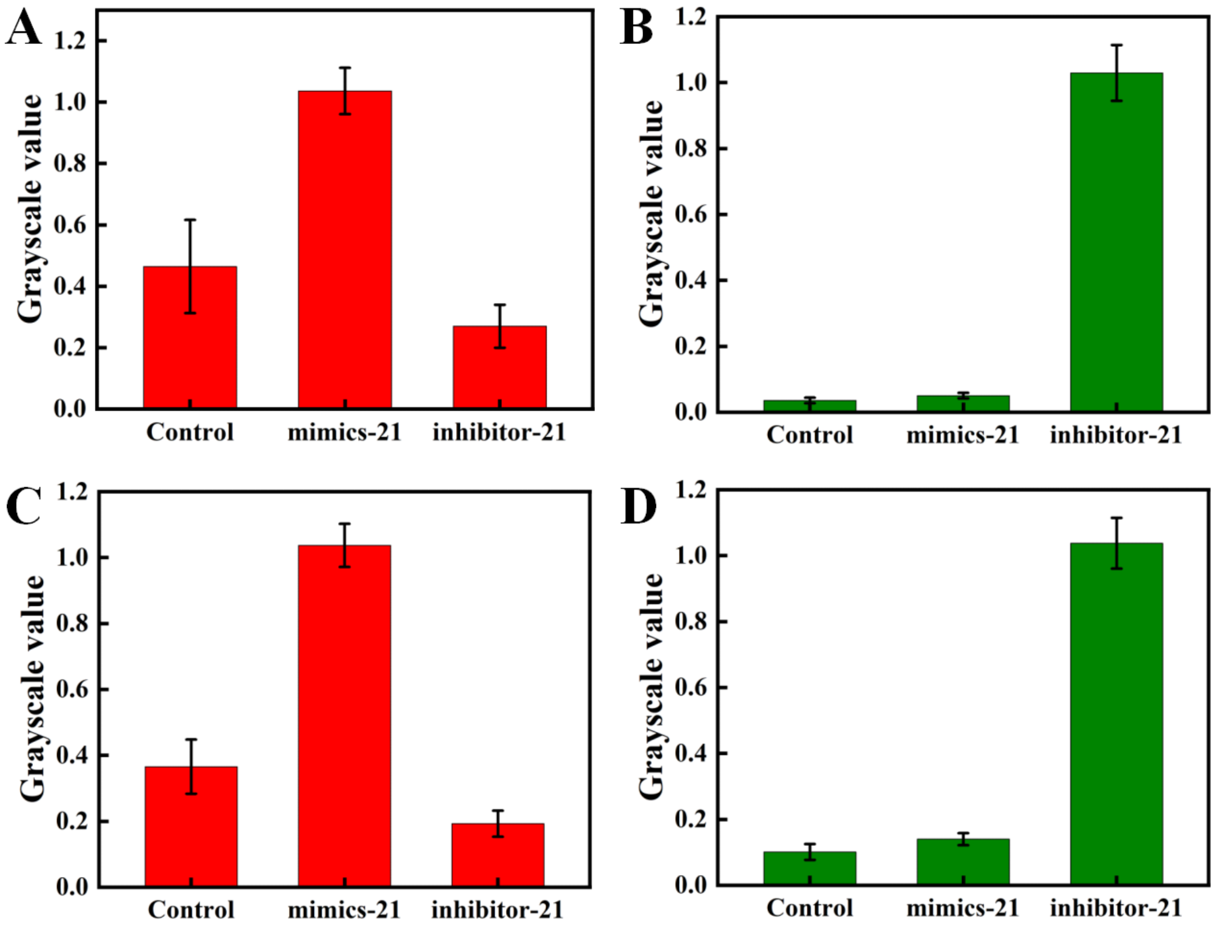

Figure S5. Grayscale values of fluorescence intensities obtained from CLSM to quantitatively reveal the different expression levels of miRNA-21 (A) and caspase-3 (B) in Hela cells after transfected with miRNA-21 mimics or inhibitor. Grayscale values of fluorescence intensities obtained from CLSM to quantitatively reveal the different expression levels of miRNA-21 (C) and caspase-3 (D) in A549 cells after transfected with miRNA-21 mimics or inhibitor.
